# Online training program maintains motor functions and quality of life in patients with Parkinson's disease

**DOI:** 10.3389/fdgth.2024.1486662

**Published:** 2024-11-13

**Authors:** Hiroshi Nakanishi, Ryoma Morigaki, Joji Fujikawa, Hiroshi Ohmae, Keisuke Shinohara, Nobuaki Yamamoto, Yuishin Izumi, Yasushi Takagi

**Affiliations:** ^1^ Department of Neurosurgery, Graduate School of Biomedical Sciences, Tokushima University, Tokushima, Japan; ^2^ Department of Research and Development, Beauty Life Corporation, Nagoya, Japan; ^3^ Parkinson’s Disease and Dystonia Research Center, Tokushima University Hospital, Tokushima, Japan; ^4^ Department of Advanced Brain Research, Graduate School of Biomedical Sciences, Tokushima University, Tokushima, Japan; ^5^ Division of Rehabilitation, Tokushima University Hospital, Tokushima, Japan; ^6^ Department of Neurology, Graduate School of Biomedical Sciences, Tokushima University, Tokushima, Japan

**Keywords:** Parkinson’s disease, telerehabilitation, online system, cognitive training, physical exercise, motor function, quality of life, frailty

## Abstract

**Objective:**

Several systematic reviews have shown that physical exercise positively affects motor function (MF) and quality of life (QoL) in patients with Parkinson's disease (PD). After the coronavirus disease (COVID-19) pandemic, numerous studies were conducted to reveal the effects of telerehabilitation for patients with PD. However, only a few empirical results of online programs for PD patients have been reported. Therefore, this study aimed to determine the effects of an online physical and cognitive training program on MF and QoL in patients with PD.

**Methods:**

We evaluated the impact of our online program on the QoL and MF of patients with PD by comparing data at baseline and after six months of intervention. For the QoL assessment, we used the Schwab and England Activities of Daily Living scale and Parkinson's Disease Questionnaire (PDQ-39), whereas, for MF, we measured movement status using the modified 20-m walk test and timed up-and-go (TUG) test.

**Results:**

We enrolled 20 patients for QoL and 19 for MF in this study. For PDQ-39, social support (*p* = 0.046, *δ* = 0.320) and cognitions (*p* = 0.028, *δ* = 0.268) significantly improved. Additionally, cadence (*p* = 0.032, *g* = −0.377) in the modified 20-m walk and exam duration (*p* = 0.003, *δ* = 0.296) and forward gait (*p* = 0.003, *δ* = 0.341) in the TUG test showed significant differences before and after the intervention.

**Conclusion:**

Our results suggest that online physical and cognitive training programs positively affect MF and QoL in individuals with PD.

## 1 Introduction

Parkinson's disease (PD) is a neurodegenerative disorder that affects motor function (MF) and non-MF in patients (1). In addition to the cardinal symptoms, such as akinesia, bradykinesia, tremor, and rigidity, patients with PD exhibit further motor deficits, including gait disturbance, impaired handwriting, reduced grip strength, and speech impairments (2). These motor deficits substantially deteriorate the quality of life (QoL) of patients with PD. Moreover, PD encompasses non-motor symptoms, e.g., olfactory loss, sleep disturbances, autonomic dysfunction, psychiatric disorders, and cognitive impairment. These may also affect QoL in patients with PD (3). While pharmacological and device-assisted therapies are widely used for managing PD (3–7), physiotherapy also plays a crucial role (3, 8, 9). Several systematic reviews have demonstrated that physical exercise positively influences MF and QoL in patients with PD (10–13). Following the coronavirus disease pandemic, there has been a considerable proliferation of scientific literature investigating the use and implementation of digital health technology (14, 15). Concurrently, numerous studies have underscored the importance of telerehabilitation for patients with PD (16–19). However, only a few empirical results of online programs for PD patients have been reported. Therefore, in this study, we aimed to evaluate the effects of an online physical and cognitive training program on MF and QoL in patients with PD.

## 2 Methods

### 2.1 Participants

This prospective study included participants with idiopathic PD ranging from stage I to stage III on the Hoehn and Yahr (H&Y) rating scale. These participants were recruited from Tokushima University Hospital between December 2021 and December 2023. Written informed consent was obtained from all participants in accordance with the Declaration of Helsinki and the Ethics Committee of Tokushima University Hospital (approval number: 4114). Patients could choose their attendance and frequency with this program. For the sample size calculation, we estimated that 24 participants would provide 80% power (with a 5% probability of type I error) and a 60% effect size using G*Power 3.1. Therefore, we set the target sample size at 24 participants.

### 2.2 Interventions

The online exercise program was broadcast via the Zoom application (Zoom Video Communications, Inc., USA) for 1 h (from 9 to 10 am), Monday to Friday, excluding national holidays. Participants could also attend the exercise program in person at our salon in Tokushima University Hospital from Tuesday to Friday. Attendance was voluntary. This program aimed to improve the QoL of participants and develop functional capabilities, such as aerobic capacity, flexibility, upper and lower limb strength, motor condition, and balance. The program comprised five parts: (1) opening session: a talk about daily topics; (2) physical exercise: stretching and resistance training; (3) cognitive training: calculation, tongue twisters, quizzes, and lectures on frailty, muscle, brain and nutrition; (4) additional exercises: bicycling, aerobic exercise on a chair, dual-task training, oral training, facial yoga, and karaoke; and (5) closing session. The opening and closing sessions took approximately 10 min and included interactive conversations and discussion about today's weather, what day it is today, and other daily topics for managing social frailty. The physical exercise took approximately 15 min and included basic stretching for warming up and resistance training for the trunk and lower body. Cognitive training took approximately 15 min and focused on stimulating cognitive functions and increasing knowledge about frailty to motivate patients. Additional exercise took approximately 15 min and changed daily to keep the program fresh. The fitness bike was used for bicycling, and aerobic exercise on a chair focused on the steps and hand movement with an up-tempo rhythm. Dual-task training included cognitive training with steps, performing different movements with the right and left hands every day for 5–10 min. Sometimes, we did karaoke during aerobic exercise with the fitness bike. Oral training was designed to maintain the functions of eating, swallowing, and speaking. Facial yoga involved forming various expressions to improve facial expressions and complexion. During the karaoke sessions, we chose songs that evoked nostalgia, with the additional aim of stimulating cognitive functions. Overall, the contents of the program were carefully selected to prevent four frailties (physical, mental, social, and oral) and scheduled daily to ensure that the overall program was engaging for participants. Given the risk of falls, all participants were required to sit on a chair and maintain a seated position throughout the program.

### 2.3 Assessment

For the primary study, patients were evaluated at baseline (T0) and after six months of intervention (T1). For the supplementary study, evaluations included results after 12 months of intervention (T2). QoL and MF were assessed. All assessments were conducted at the salon in Tokushima University Hospital, although some questionnaires for QoL were administered by telephone when participants could not visit the salon. The inclusion criteria were as follows: (i) a diagnosis of idiopathic PD, (ii) stages I to III on the H&Y rating scale, (iii) the ability to exercise while seated, and (iv) age older than 30 years. The exclusion criteria included the following: (i) lack of patient consent, (ii) being deemed inappropriate for participation, and (iii) having attended fewer than six practice sessions over 6 months.

### 2.4 Outcomes

#### 2.4.1 Quality of life (QoL)

To evaluate the impact of our program on QoL, we employed the Schwab and England Activities of Daily Living (S&E-ADL) scale and the Japanese version of the Parkinson's Disease Questionnaire (PDQ-39) (20, 21). The S&E-ADL scale uses percentages to indicate the patient's level of independence in daily activities, with 100% representing complete independence and 0% representing full dependence. The PDQ-39 comprises a 39-item questionnaire encompassing eight distinct dimensions. Participants completed the questionnaires independently; however, responses were recorded via telephone for those unable to visit the salon.

#### 2.4.2 Motor function (MF)

**Figure 1:**
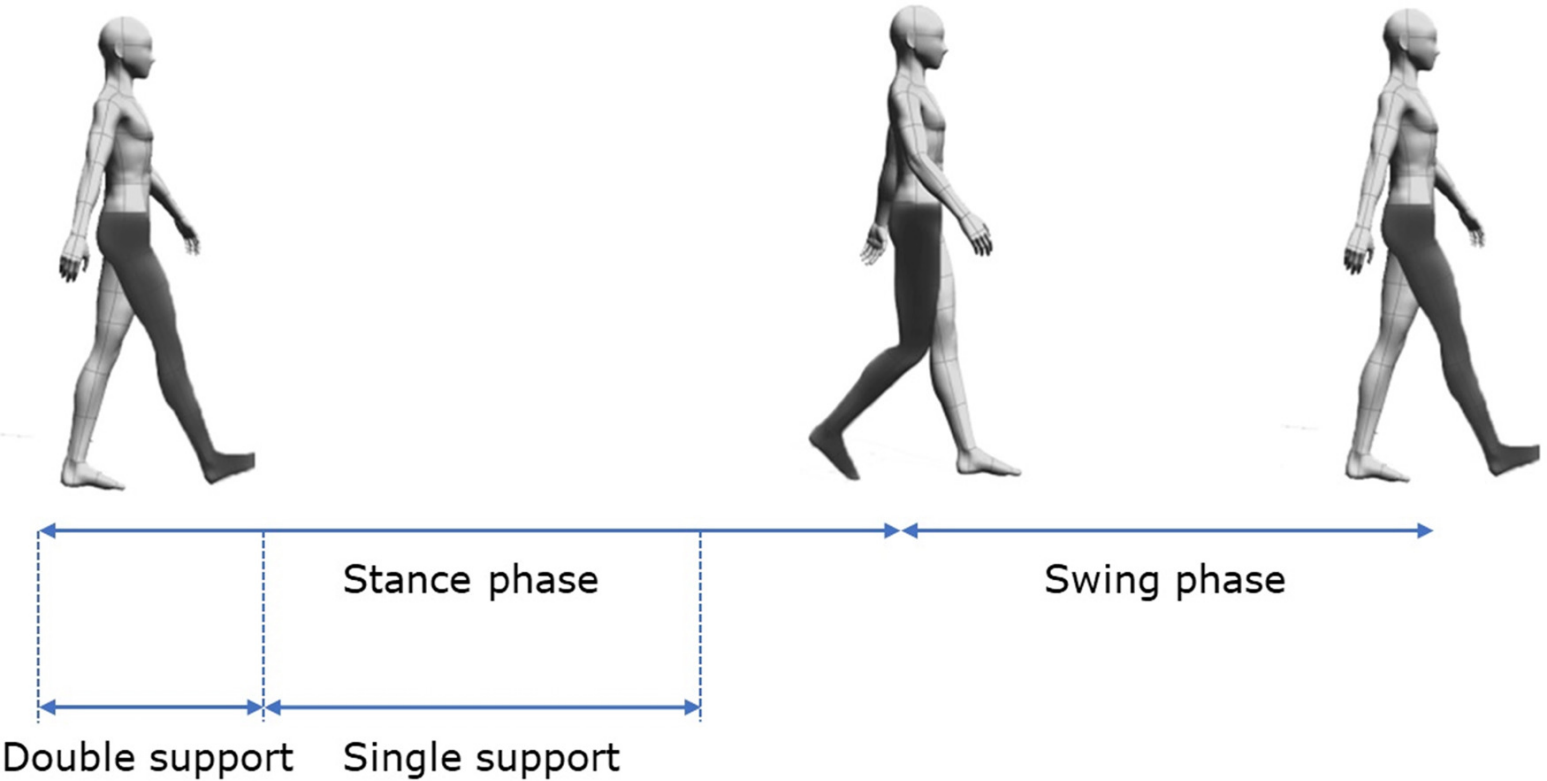
Illustration of gait cycle phases in the modified 20-m walk test.

To assess MF, we evaluated movement using the modified 20-m walk test and the timed up-and-go (TUG) test. Both tests used the wireless inertial sensor system BTS G-WALK (BTS Bioengineering S.p.A., Italy) to measure spatiotemporal parameters and phase durations. In the modified 20-m walk test, participants walked 10 m forward, turned around, and returned to the starting point. The BTS G-WALK assessed results using data from the forward and return walks. In the TUG test, participants began seated, stood up, walked to a target 3 m away, turned around the target, returned to the chair, turned, and sat down. The parameters measured during the modified 20-m walk test included the following: (1) gait cycle phases (walk quality index and phase percentages for stance, swing, double support, and single support, for both less affected and more affected sides); (2) spatiotemporal parameters (global parameters, such as cadence and speed, and differentiated parameters, such as stride length and step length for both less affected and more affected sides); and (3) symmetry and propulsion indices. Due to the asymmetrical nature of PD, the more affected and less affected sides were determined through a combination of patient self-reporting and clinical assessment by the physician. Gait cycle phases are illustrated in Figure 1.

**Figure 2:**
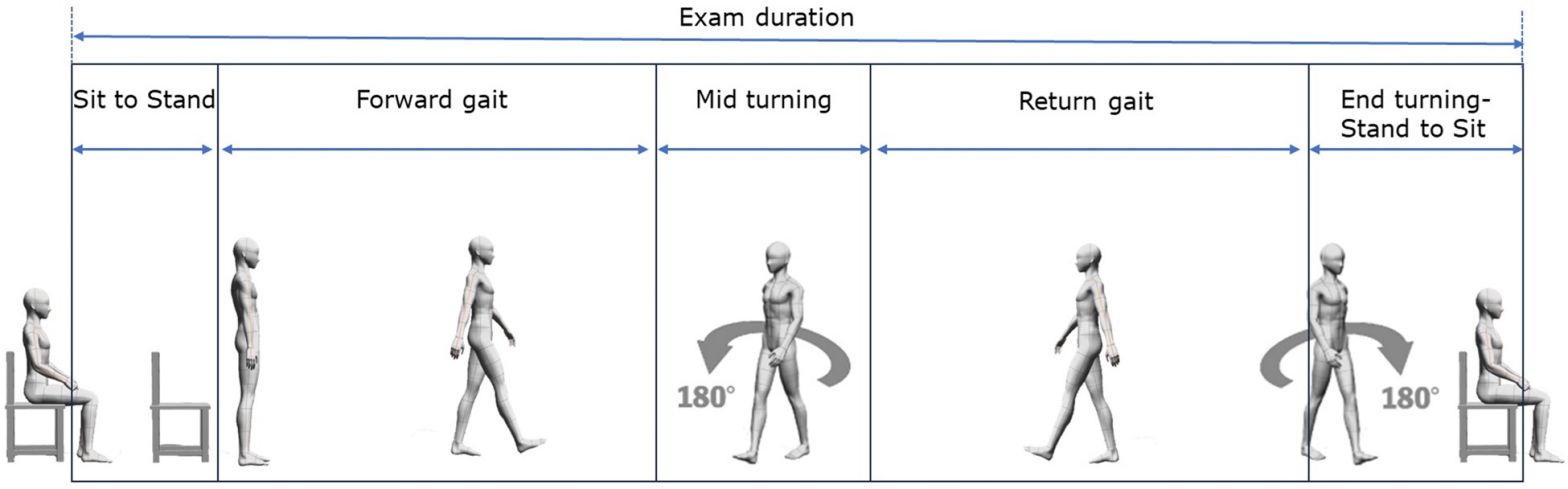
Description of phase durations in the timed up-and-go test.

The parameters measured from the TUG test included the following: (1) spatiotemporal parameters: (a) for “sit to stand” and “stand to sit” phases, we measured phase duration, anterior-posterior acceleration, lateral acceleration, and vertical acceleration; (b) for “mid turning” and “end turning” phases, we measured phase duration, maximum rotation speed, and average rotation speed; and (2) overall phase duration for “sit to stand”, “forward gait”, “mid turning”, “return gait”, “end turning-stand to sit”, and “exam duration”. The phase durations are described in Figure 2.

### 2.5 Statistical analysis

All measured data are presented as mean ± standard error. For QoL analysis, Wilcoxon's signed-rank test was conducted to assess the significance between the two groups (T0 and T1), and the effect size was evaluated using Cliff's delta. For supplementary analysis, Friedman's test was applied among the three groups (T0, T1, and T2). A *post hoc* analysis using Wilcoxon's signed-rank test was performed when significance was found. For MF analysis, the paired *t*-test was used to analyze the two groups (T0 and T1) when the normality of distribution was verified by Shapiro–Wilk's test. If normality was not confirmed, Wilcoxon's signed-rank test was employed. Significance and effect size were determined using Hedges' g when normality was observed or Cliff's delta when it was not. For supplementary analysis of the three groups (T0, T1, and T2), the normality of distribution was first verified by Shapiro–Wilk's test. If normality was not observed, Friedman's test was performed. When normality was observed, sphericity was assessed with Mendoza's multi-sample sphericity test. One-way repeated measures analysis of variance (rANOVA) was performed without adjustment if sphericity was observed; if not, rANOVA was conducted with adjustment using the lower bound of epsilon (*ε*). *Post hoc* analyses were conducted using Wilcoxon's signed-rank test when Friedman's test showed significance and the paired *t*-test when rANOVA showed significance. Bonferroni's correction was applied for all *post hoc* tests. All statistical analyses were performed using R (version 4.2.1) (22), and the significance level was set at *p* < 0.05.

## 3 Results

### 3.1 Participant characteristics

For the primary study (T0 and T1), 20 patients for QoL and 19 for MF were enrolled and evaluated on the basis of the inclusion and exclusion criteria. For QoL, 23 patients were initially recruited, but three patients with attendance of less than 6 days were excluded from the study. Similarly, 20 patients were recruited for MF, with one subsequently excluded. The number of patients, their characteristics, and attendance results in the QoL and MF groups are displayed in Table 1. For the QoL assessment, the average age was 70.00 (±6.31) years, average total attendance over 6 months was 32.85 (±27.48) days, and online attendance rate was 63.6%. The mean monthly attendance was 5.48 (±4.58), while the median monthly attendance was 5.10 (±4.64). For the MF assessment, the average age was 66.74 (±8.67) years, average total attendance over 6 months was 37.58 (±25.27) days, and online attendance rate was 70.0%. The mean monthly attendance was 6.26 (±4.21), while the median monthly attendance was 5.87 (±4.46).

**Table 1:** Participant characteristics in the quality of life and motor function groups (6 months).

Characteristic	*n* = 20 (QoL)	*n* = 19 (MF)
Age, years (mean ± SD)	70.00 ± 6.31	66.74 ± 8.67
Sex, *n* (%)		
Female	13 (65%)	12 (63%)
Male	7 (35%)	7 (37%)
Hoehn and yahr stage, *n* (%)		
I	9 (45%)	9 (47%)
II	5 (25%)	4 (21%)
III	6 (30%)	6 (32%)
Number of attendances, days (mean ± SD)		
Offline	11.95 ± 6.75	11.26 ± 7.01
Online	20.90 ± 30.86	26.32 ± 29.33
Overall	32.85 ± 27.48	37.58 ± 25.27
Average monthly attendances, days		
Mean (mean ± SD)	5.48 ± 4.58	6.26 ± 4.21
Median (mean ± SD)	5.10 ± 4.64	5.87 ± 4.46

QoL, quality of life; MF, motor function; SD, standard deviation.

For the supplementary study (T0, T1, and T2), 10 patients for QoL and nine for MF were enrolled and evaluated on the basis of the inclusion and exclusion criteria. For the QoL assessment, the average age was 67.50 (±4.79) years, average total attendance over 12 months was 88.80 (±60.16) days, and online attendance rate was 69.6%. The mean monthly attendance was 7.40 (±5.01), while the median monthly attendance was 6.55 (±5.31). For the MF assessment, the average age was 65.67 (±8.56) years, average total attendance over 12 months was 110.89 (±54.32) days, and online attendance rate was 81.9%. The mean monthly attendance was 9.24 (±4.53), while the median monthly attendance was 9.44 (±4.82).

### 3.2 QoL for T0 and T1

The results of the QoL assessment are presented in Table 2. No significant difference in activity of daily living (ADL) was found using the S&E-ADL. However, significant differences were observed in the PDQ-39 for the following items: mobility (*p* = 0.021, *δ* = −0.155), social support (*p* = 0.046, *δ* = 0.320), and cognitions (*p* = 0.028, *δ* = 0.268). Among these three items, social support and cognitions had positive effect sizes, indicating that participants felt better after the program despite the small effect sizes.

**Table 2:** Result of the quality of life test (6 months).

Variable	T0 (Mean ± SE)	T1 (Mean ± SE)	*p*-value^a^	Effect size^b^
S&E-ADL				
Rating, %	71.75 ± 3.65	71.00 ± 4.10	0.685	0.013
PDQ-39				
Mobility	26.85 ± 2.22	28.90 ± 2.21	**0**.**021**	−0.155
Activities of daily living	14.75 ± 1.23	16.60 ± 1.43	0.080	−0.160
Emotional well-being	16.15 ± 1.17	15.10 ± 1.22	0.081	0.128
Stigma	9.25 ± 0.85	8.30 ± 0.76	0.231	0.175
Social support	7.25 ± 0.71	5.60 ± 0.48	**0**.**046**	0.320
Cognitions	10.35 ± 0.76	8.85 ± 0.73	**0**.**028**	0.268
Communication	6.85 ± 0.66	5.95 ± 0.48	0.096	0.163
Bodily discomfort	7.60 ± 0.67	6.50 ± 0.47	0.064	0.245

The values of T0 and T1 are compared. T0, at baseline; T1, after 6 months of intervention; S&E-ADL, Schwab and England Activities of Daily Living scale; PDQ-39, Japanese version of the Parkinson's Disease Questionnaire; SE, standard error.

^a^ Wilcoxon's signed-rank test.

^b^ Cliff's delta (small size ≥ 0.147, medium size ≥ 0.330, large size ≥ 0.474).

Bold values denote statistical significance at the *p* < 0.05 level.

### 3.3 MF for T0 and T1

The results of the MF assessment are shown in Tables 3, 4. For the modified 20-m walk test, a statistically significant difference was found in cadence (*p* = 0.032, *g* = −0.377), although the effect size was small. In the TUG test, significance was found in two items: exam duration (*p* = 0.003, *δ* = 0.296) and forward gait (*p* = 0.003, *δ* = 0.341). Both items showed positive effect sizes, with forward gait demonstrating a medium-sized effect.

**Table 3:** Result of the modified 20-m walk test (6 months).

Variable	T0 (Mean ± SE)	T1 (Mean ± SE)	*p*-value	Effect size
1. Gait cycle phases				
Less affected side				
Walk quality index, %	96.23 ± 0.62	96.51 ± 0.87	0.444^b^	−0.175^d^
Stance phase, % cycle	60.75 ± 0.51	60.59 ± 0.58	0.709^a^	0.065^c^
Swing phase, % cycle	39.25 ± 0.51	39.41 ± 0.58	0.709^a^	−0.065^c^
Double support, % cycle	10.19 ± 0.52	10.46 ± 0.53	0.444^a^	−0.112^c^
Single support, % cycle	39.97 ± 0.69	39.84 ± 0.62	0.663^a^	0.094^c^
More affected side				
Walk quality index, %	95.42 ± 0.92	91.09 ± 4.70	0.904^b^	−0.030^d^
Stance phase, % cycle	60.09 ± 0.71	60.16 ± 0.65	0.882^a^	−0.023^c^
Swing phase, % cycle	39.91 ± 0.71	39.84 ± 0.65	0.882^b^	0.023^d^
Double support, % cycle	10.63 ± 0.54	10.51 ± 0.44	0.778^b^	0.053^d^
Single support, % cycle	39.34 ± 0.56	39.33 ± 0.56	0.970^a^	0.007^c^
2. Spatio-temporal parameters				
(a) Global parameters				
Cadence, steps/min	116.12 ± 3.18	121.67 ± 3.43	**0**.**032**^a^	−0.377^c^
Speed, m/s	1.08 ± 0.05	1.16 ± 0.06	0.092^a^	−0.319^c^
(b) Differentiated parameters				
Less affected side				
Stride length, m	1.12 ± 0.04	1.15 ± 0.03	0.497^b^	−0.158^d^
Step length, % stride length	50.70 ± 0.73	50.83 ± 0.49	0.855^a^	−0.048^c^
More affected side				
Stride length, m	1.12 ± 0.04	1.15 ± 0.03	0.332^a^	−0.196^c^
Step length, % stride length	49.30 ± 0.73	49.17 ± 0.49	0.855^a^	0.048^c^
3. Symmetry and propulsion indices				
Symmetry index, %	90.85 ± 1.73	91.16 ± 1.56	0.904^b^	0.006^d^
Propulsion index, %				
Less affected side	6.83 ± 0.66	7.79 ± 0.68	0.136^b^	−0.219^d^
More affected side	6.86 ± 0.66	8.10 ± 0.79	0.108^a^	−0.382^c^

The values of T0 and T1 are compared. Stance phase + Swing phase = 100%. T0, at baseline; T1, after 6 months of intervention; SE, standard error.

^a^ Paired *t*-test.

^b^ Wilcoxon's signed-rank test.

^c^ Hedges's g (small size ≥ 0.2, medium size ≥ 0.5, large size ≥ 0.8).

^d^ Cliff's delta (small size ≥ 0.147, medium size ≥ 0.330, large size ≥ 0.474).

Bold value denotes statistical significance at the *p* < 0.05 level.

**Table 4:** Results of the timed up-and-go test (6 months).

Variable	T0 (Mean ± SE)	T1 (Mean ± SE)	*p*-value	Effect size
1. Spatio-temporal parameters				
(a) Parameters for “sit to stand” and “stand to sit"				
Sit to stand				
Anterio-posterior acceleration, m/s^2^	4.23 ± 0.80	3.90 ± 0.42	0.679^b^	−0.053^d^
Lateral acceleration, m/s^2^	2.21 ± 0.39	2.26 ± 0.23	0.349^b^	−0.188^d^
Vertical acceleration, m/s^2^	4.50 ± 0.62	5.27 ± 0.47	0.210^a^	−0.318^c^
Stand to sit				
Anterio-posterior acceleration, m/s^2^	5.42 ± 0.71	5.20 ± 0.67	0.891^b^	0.122^d^
Lateral acceleration, m/s^2^	3.56 ± 0.40	3.79 ± 0.50	0.683^a^	−0.116^c^
Vertical acceleration, m/s^2^	6.95 ± 0.58	7.55 ± 0.82	0.514^a^	−0.191^c^
(b) Parameters for “mid turning” and “end turning”				
Mid turning				
Maximum rotation speed, °/s	159.21 ± 14.11	144.18 ± 10.78	0.164^a^	0.269^c^
Average rotation speed,°/s	84.01 ± 6.86	76.61 ± 5.17	0.147^a^	0.274^c^
End turning				
Maximum rotation speed, °/s	168.15 ± 12.46	172.08 ± 12.98	0.640^a^	−0.070^c^
Average rotation speed, °/s	84.95 ± 8.12	94.72 ± 8.44	0.191^a^	−0.265^c^
2. Phase durations				
Sit to stand, s	1.65 ± 0.17	1.40 ± 0.09	0.145^a^	0.396^c^
Forward gait, s	4.12 ± 0.73	2.57 ± 0.32	**0**.**003**^b^	0.341^d^
Mid turning, s	2.56 ± 0.37	2.52 ± 0.24	0.465^b^	−0.155^d^
Return gait, s	3.52 ± 0.76	2.11 ± 0.28	0.059^b^	0.202^d^
End turning - stand to sit, s	3.20 ± 0.40	2.77 ± 0.31	0.225^b^	0.194^d^
Exam duration, s	16.02 ± 1.87	11.61 ± 0.89	**0**.**003**^b^	0.296^d^

The values of T0 and T1 are compared. T0, at baseline; T1, after 6 months of intervention; SE, standard error.

^a^ Paired *t*-test.

^b^ Wilcoxon's signed-rank test.

^c^ Hedges's g (small size ≥ 0.2, medium size ≥ 0.5, large size ≥ 0.8).

^d^ Cliff's delta (small size ≥ 0.147, medium size ≥ 0.330, large size ≥ 0.474).

Bold values denote statistical significance at the *p* < 0.05 level.

### 3.4 QoL for T0, T1, and T2

No statistically significant differences were found in ADL measured by the S&E-ADL. For the eight dimensions of the PDQ-39, significance was noted only for ADL in the PDQ-39 by Friedman's test, although *post hoc* analysis was not feasible.

### 3.5 MF for T0, T1, and T2

For the modified 20-m walk test, we found significant differences for cadence (*p* = 0.003), speed (*p* = 0.013), and symmetry index (*p* = 0.050) by Friedman's test, and we also found significant differences for cadence (*p* = 0.008, *δ* = −0.506) and speed (*p* = 0.008, *δ* = −0.358) by *post hoc* analysis of T0 and T1 and for symmetry index (*p* = 0.012, *δ* = 0.383) of T0 and T2. For TUG test results, no statistically significant difference was found.

### 3.6 Adverse events

No adverse events were observed among participants during the program.

## 4 Discussion

This is the first study to demonstrate the effects of an online physical and cognitive training program on MF and QoL in Japanese patients with PD. Our findings suggest that online programs positively impact MF and QoL in this population. Specifically, we observed a significant improvement in cadence during the modified 20-m walk and in the exam duration and forward gait phase of the TUG test over 6 months. These results align with those in previous research indicating that physical exercise enhances MF in patients with PD. Furthermore, our study revealed a statistically significant enhancement in social support and cognitions measured by the PDQ-39 over 6 months, supporting the past research findings showing that physical exercise may benefit cognitive function in patients with PD (23). Aerobic exercises, including bicycling and chair-based aerobic exercises implemented in our program, were particularly beneficial for MF and cognitive function, consistent with results of existing literature (10, 24–26). Additionally, dual-task exercises in our program likely contributed positively to motor and cognitive function improvement among the participants (27–29).

In our program, all exercises were conducted in a seated position, including those performed during in-person sessions. This approach demonstrated a notable safety profile, as we observed no adverse events throughout the study period. However, we acknowledge that this seated methodology may have imposed certain limitations on gait, mobility, and balance training. Despite this potential constraint, it is important to note that existing literature supports the efficacy of seated exercises in improving gait, mobility, and balance outcomes in older adults (30–33). In patients with PD, one study showed the effect of a sitting-based dance class for balance (34). These studies imply the possibility that seated exercises have positive effects on gait, mobility, and balance for patients with PD.

To maintain participant engagement and motivation, we incorporated various additional exercise sessions, such as oral exercises, facial yoga, karaoke, and enhanced communication among attendees (35). Educational sessions on frailty were also integrated into our program to underscore the importance of daily exercise, potentially bolstering participant motivation. Furthermore, use of incentive-based behavioral therapies is likely to be effective for PD (36). The engagement and excitement generated during dual-task training may potentially stimulate dopamine release, suggesting that this approach could be effective not only for cognitive and motor improvements but also for enhancing motivation in patients with PD. The dopaminergic activation associated with the engaging nature of dual-task exercises may contribute to increased motivation, potentially leading to improved adherence and outcomes in rehabilitation programs (37, 38). Further research is warranted to elucidate whether dual-task training influences motivation or not and quantify its effects on long-term patient engagement and therapeutic efficacy.

Although the number of patients was insufficient, worsening of the symmetry index in the modified 20-m walk test from baseline to 12 months was found. This might reflect the aggravation in asymmetric posture of PD. Axial postural abnormalities are known to aggravate in PD, with lateral abnormalities specifically recognized as Pisa syndrome (39). While the exact etiology remains unclear, there is growing evidence suggesting the involvement of impaired sensory-motor integration (40). Interventions stimulating sensory-motor integration, such as dual-task training, could be beneficial and increasing the duration and frequency of these exercises could potentially prevent the aggravation of postural asymmetry (41). Besides the symmetry index, we did not observe sustained effects of our program beyond 12 months, with improvements regressing to baseline levels after 6 months, indicating no worsening of symptoms over 12 months. It is plausible that the efficacy of exercise interventions for PD may plateau within a year. Given the progressive nature of PD, our results suggest a potential positive impact of our program.

Several studies have demonstrated the effectiveness of remotely supervised exercise and telerehabilitation for patients with PD (42–45). Telerehabilitation benefits patients with PD and enhances participation among frail older individuals by leveraging online platforms, which reduces barriers to engagement, as illustrated by our findings.

Regarding the limitations of this study, first, for MF assessment, we assessed only supervised gait and mobility which might not predict the actual real-world mobility correctly. In addition, we did not measure freezing of gait (FoG) scores, but there is a possibility that FoG could affect our results, especially parameters for forward gait, mid turning, and end turning. Moreover, for the cognitive evaluation, even though the PDQ-39 included scales about cognitions, it was subjective and we did not perform an objective one. Additionally, we set no exclusion criteria for severely cognitively impaired patients. These points should be considered when evaluating the effect shown in this research. Furthermore, we did not force patients to attend the program periodically or set the number of participations, so participation frequency varied among patients and we did not analyze the impact of participation frequency in this study, although most patients attended the program roughly evenly. We also recognize that the influence of online exercise programs is constrained and that distinguishing the effects of our program from other factors was not feasible. We did not differentiate the effects of individual exercise components and sessions. Finally, we did not distinguish between the effects of offline and online program formats. Despite these limitations, our program can positively influence participants’ motivation to exercise and their daily activity levels.

## 5 Conclusions

We evaluated the impact of an online program on the QoL and MF of patients with PD by comparing data at baseline and after six months of intervention. The QoL assessment was subjective, but significant differences were observed in mobility, social support, and cognitions in the PDQ-39. The MF assessment was objective, and significant differences were observed in cadence of the modified 20-m walk test and exam duration and forward gait in the TUG test. Although PD is a progressive disease and is not easy to improve, our results suggest that an online physical and cognitive training program has positive effects on MF and QoL for people with PD.

## Data Availability

The original contributions presented in the study are included in the article/Supplementary Material, further inquiries can be directed to the corresponding author.
