# Correction: Online training program maintains motor functions and quality of life in patients with Parkinson's disease

**DOI:** 10.3389/fdgth.2025.1672261

**Published:** 2025-08-05

**Authors:** Hiroshi Nakanishi, Ryoma Morigaki, Joji Fujikawa, Hiroshi Ohmae, Keisuke Shinohara, Nobuaki Yamamoto, Yuishin Izumi, Yasushi Takagi

**Affiliations:** ^1^ Department of Neurosurgery, Graduate School of Biomedical Sciences, Tokushima University, Tokushima, Japan; ^2^ Department of Research and Development, Beauty Life Corporation, Nagoya, Japan; ^3^ Parkinson’s Disease and Dystonia Research Center, Tokushima University Hospital, Tokushima, Japan; ^4^ Department of Advanced Brain Research, Graduate School of Biomedical Sciences, Tokushima University, Tokushima, Japan; ^5^ Division of Rehabilitation, Tokushima University Hospital, Tokushima, Japan; ^6^ Department of Neurology, Graduate School of Biomedical Sciences, Tokushima University, Tokushima, Japan

**Keywords:** Parkinson's disease, telerehabilitation, online system, cognitive training, physical exercise, motor function, quality of life, frailty

There was an error in the description of the statistical method. The analysis results remain unaffected, as the correct statistical method has already been applied.

Corrections have been made to the section Methods, Statistical analysis:

All measured data are presented as mean ± standard error. For QoL analysis, Wilcoxon's signed-rank test was conducted to assess the significance between the two groups (T0 and T1), and the effect size was evaluated using Cliff's delta. For supplementary analysis, Friedman's test was applied among the three groups (T0, T1, and T2). A *post hoc* analysis using Wilcoxon's signed-rank test was performed when significance was found. For MF analysis, the paired *t*-test was used to analyze the two groups (T0 and T1) when the normality of distribution was verified by Shapiro–Wilk's test. If normality was not confirmed, Wilcoxon's signed-rank test was employed. Significance and effect size were determined using Hedges' g when normality was observed or Cliff's delta when it was not. For supplementary analysis of the three groups (T0, T1, and T2), the normality of distribution was first verified by Shapiro–Wilk's test. If normality was not observed, Friedman's test was performed. When normality was observed, sphericity was assessed with Mendoza's multi-sample sphericity test. One-way repeated measures analysis of variance (rANOVA) was performed without adjustment if sphericity was observed; if not, rANOVA was conducted with adjustment using the lower bound of epsilon (*ε*). *Post hoc* analyses were conducted using Wilcoxon's signed-rank test when Friedman's test showed significance and the paired *t*-test when rANOVA showed significance. Bonferroni's correction was applied for all *post hoc* tests. All statistical analyses were performed using R (version 4.2.1) (22), and the significance level was set at *p* < 0.05.

In the article the same sentence is repeated twice, “For TUG test results, no statistically significant difference was found. For the TUG test results, no statistically significant difference was found”.

A correction has been made to Results, 3.5 MF for T0, T1, and T2:

“For TUG test results, no statistically significant difference was found.”

The original version of this article has been updated.

