# Supplementary material for: Online training program maintains motor functions and quality of life in patients with Parkinson's disease
Source: Front Digit Health. 2024 Nov 13;6:1486662. doi: 10.3389/fdgth.2024.1486662 (PMC11599239; doi:10.3389/fdgth.2024.1486662)
Supplement: Supplementary file 1 [file Table1.pdf]

Table S1. Items measured for the modified 20m walk test.

Items	Unit	Description
**1. Gait cycle phases**		
Walk quality index (L/M)	%	Ability to divide his gait cycle correctly between support phase and swing phase
Stance phase (L/M)	% cycle	Average value of the durations of the right and left stance phases as percentages of the duration of the gait cycle
Swing phase (L/M)	% cycle	Average value of the durations of the right and left swing phases as percentages of the duration of the gait cycle
Double support (L/M)	% cycle	Average value of the durations of the right and left support phases as percentages of the duration of the gait cycle
Single support (L/M)	% cycle	Average value of the single right and left support phases as percentages of the duration of the gait cycle
**2. Spatio-temporal parameters**		
(a) Global parameters		
Cadence	steps/min	Number of half-steps in one minute
Speed	m/s	Average speed of the walk
(b) Differentiated parameters		
Stride length (L/M)	m	Mean value of the distances between each initial contact and the next of the same foot
Step length (L/M)	% stride length	Average value of distances between each initial contact and the next one of the contralateral side
**3. Symmetry and propulsion indices**		
Symmetry index	%	Ability to accelerate the center of mass in a similar way during the cycle of the right and left steps
Propulsion index (L/M)	%	Ability to fully accept body weight on a limb after the deceleration phase and push the center of mass forward on the opposite limb (acceleration phase)

L: less affected side, M: more affected side.
