# Supplementary material for: Online training program maintains motor functions and quality of life in patients with Parkinson's disease
Source: Front Digit Health. 2024 Nov 13;6:1486662. doi: 10.3389/fdgth.2024.1486662 (PMC11599239; doi:10.3389/fdgth.2024.1486662)
Supplement: Supplementary file 2 [file Table2.pdf]

Table S2. Items measured for the timed up and go test.

Items	Unit	Description
**1. Spatio-temporal parameters**		
(a) Parameters for "sit to stand" and "stand to sit"		
Phase duration	s	Average value of the time interval for each movement
Anterio-posterior acceleration	m/s^2	Average value of the antero-posterior acceleration range reached during each assessed phase
Lateral acceleration	m/s^2	Average value of the medial lateral acceleration range reached during each assessed phase
Vertical acceleration	m/s^2	Average value of the vertical acceleration range reached during each assessed phase
(b) Parameters for "mid turning" and "end turning"		
Phase duration	s	Average value of the temporal duration of each turn in the test
Maximum rotation speed	°/s	Maximum speed reached in each turn
Average rotation speed	°/s	Average speed during each turn
**2. Phases duration**		
Sit to stand	s	Duration of sit to stand phase
Forward gait	s	Duration of forward gait phase
Mid turning	s	Duration of mid turning phase
Return gait	s	Duration of return gait phase
End turning - stand to sit	s	Duration of end turning - stand to sit phase
Exam duration	s	Total duration of examination
