# Supplementary material for: Online training program maintains motor functions and quality of life in patients with Parkinson's disease
Source: Front Digit Health. 2024 Nov 13;6:1486662. doi: 10.3389/fdgth.2024.1486662 (PMC11599239; doi:10.3389/fdgth.2024.1486662)
Supplement: Supplementary file 3 [file Table3.pdf]

Table S3. Participant characteristics in the quality of life and motor function groups (12 months).

Characteristics	n=10 (QoL)	n=9 (MF)
Age, years (mean ± SD)	67.50 ± 4.79	65.67 ± 8.56
Sex, n (%)		
Female	7 (70%)	6 (67%)
Male	3 (30%)	3 (33%)
Hoehn and Yahr stage, n (%)		
I	4 (40%)	5 (56%)
II	1 (10%)	2 (22%)
III	5 (50%)	2 (22%)
Number of attendances, days (mean ± SD)		
Off-line	27.00 ± 13.17	20.11 ± 16.37
On-line	61.80 ± 63.05	90.78 ± 60.13
Total	88.80 ± 60.16	110.89 ± 54.32
Average monthly attendances, days		
Mean (mean ± SD)	7.40 ± 5.01	9.24 ± 4.53
Median (mean ± SD)	6.55 ± 5.31	9.44 ± 4.82

QoL: quality of life, MF: motor function, SD: standard deviation.
