# Supplementary material for: Online training program maintains motor functions and quality of life in patients with Parkinson's disease
Source: Front Digit Health. 2024 Nov 13;6:1486662. doi: 10.3389/fdgth.2024.1486662 (PMC11599239; doi:10.3389/fdgth.2024.1486662)
Supplement: Supplementary file 4 [file Table4.pdf]

Table S4. Result of the quality of life test (12 months)

Variable	T0 Mean ± SE	T1 Mean ± SE	T2 Mean ± SE	*p-value*
**SE-ADL**				
Rating, %	68.50 ± 5.78	68.00 ± 7.42	71.00 ± 6.74	0.720 [a]
**PDQ-39**				
Mobility	27.80 ± 3.60	29.20 ± 3.85	26.30 ± 3.79	0.303 [a]
Activities of daily living (ADL)	15.70 ± 1.62	17.50 ± 2.03	13.90 ± 2.05	0.046* [a]
Emotional well-being	16.10 ± 1.69	15.10 ± 1.80	13.80 ± 1.94	0.113 [a]
Stigma	8.50 ± 1.07	8.70 ± 1.35	8.60 ± 1.16	0.882 [a]
Social support	7.20 ± 0.85	6.30 ± 0.63	5.40 ± 0.81	0.273 [a]
Cognitions	9.00 ± 1.02	7.80 ± 0.84	6.80 ± 0.65	0.080 [a]
Communication	7.40 ± 0.99	6.10 ± 0.71	6.00 ± 0.73	0.401 [a]
Bodily discomfort	7.00 ± 0.97	6.80 ± 0.70	6.20 ± 0.79	0.823 [a]

The values of T0, T1 and T2 are compared. *: $p < 0.05$ is considered as significant. a: Friedman's test. T0: at baseline, T1: after 6 months of intervention, T2: after 12 months of intervention. S&E-ADL: the Schwab and England Activities of Daily Living scale, PDQ-39, the Japanese version of the Parkinson's Disease Questionnaire, SE: standard error.
