# Supplementary material for: Online training program maintains motor functions and quality of life in patients with Parkinson's disease
Source: Front Digit Health. 2024 Nov 13;6:1486662. doi: 10.3389/fdgth.2024.1486662 (PMC11599239; doi:10.3389/fdgth.2024.1486662)
Supplement: Supplementary file 5 [file Table5.pdf]

Table S5. Result of the modified 20m walk test (12 months).

Variable	T0 (Mean ± SE)	T1 (Mean ± SE)	T2 (Mean ± SE)	*p-value*
**1. Gait cycle phases**				
Less affected side				
Walk quality index, %	96.03 ± 0.79	96.54 ± 1.20	95.03 ± 0.70	0.097 [a]
Stance phase, % cycle	61.37 ± 0.64	60.13 ± 0.86	61.18 ± 0.85	0.204 [b]
Swing phase, % cycle	38.63 ± 0.64	39.87 ± 0.86	38.82 ± 0.85	0.206 [b]
Double support, % cycle	10.53 ± 0.86	9.81 ± 0.86	10.27 ± 0.70	0.356 [b]
Single support, % cycle	40.39 ± 0.85	40.07 ± 0.95	41.14 ± 1.14	0.302 [b]
More affected side				
Walk quality index, %	95.83 ± 1.02	85.78 ± 9.83	94.98 ± 1.60	0.459 [a]
Stance phase, % cycle	59.47 ± 0.88	59.92 ± 0.98	59.02 ± 1.14	0.401 [b]
Swing phase, % cycle	40.53 ± 0.88	40.08 ± 0.98	40.98 ± 1.14	0.401 [b]
Double support, % cycle	10.76 ± 0.54	10.62 ± 0.68	9.49 ± 1.05	0.202 [b]
Single support, % cycle	38.43 ± 0.69	39.66 ± 0.79	35.80 ± 3.68	0.319 [a]
**2. Spatio-temporal parameters**				
(a) Global parameters				
Cadence, steps/min	115.30 ± 4.65	126.62 ± 5.44[†]	124.19 ± 6.25	0.003* [a]
Speed, m/s	1.11 ± 0.08	1.27 ± 0.09[‡]	1.21 ± 0.08	0.013* [a]
(b) Differentiated parameters				
Less affected side				
Stride length, m	1.16 ± 0.05	1.20 ± 0.04	1.17 ± 0.05	0.352 [b]
Step length, % stride length	50.67 ± 1.11	50.96 ± 0.84	52.10 ± 0.94	0.279 [b]
More affected side				
Stride length, m	1.15 ± 0.06	1.20 ± 0.05	1.17 ± 0.05	0.344 [b]
Step Length, % stride length	49.33 ± 1.11	49.04 ± 0.84	47.90 ± 0.94	0.279 [b]

3. Symmetry and propulsion indices				
Symmetry index, %	93.36 ± 1.32	92.26 ± 2.55	88.84 ± 2.81§	0.050* a
Propulsion index, %				
Less affected side	6.69 ± 0.70	9.12 ± 0.96	8.83 ± 1.43	0.093 b
More affected side	7.24 ± 0.77	9.28 ± 1.18	8.93 ± 1.33	0.104 b

The values of T0, T1 and T2 are compared. Stance phase + Swing phase = 100%. *: $p < 0.05$ is considered as significant, †: $p < 0.05$ vs T0,‡: $p < 0.05$ vs T0, §: $p < 0.05$ vs T0. a: Friedman's test. b: rANOVA. T0: at baseline, T1: after 6 months of intervention, T2: after 12 months of intervention, SE: standard error.
