# Supplementary material for: Online training program maintains motor functions and quality of life in patients with Parkinson's disease
Source: Front Digit Health. 2024 Nov 13;6:1486662. doi: 10.3389/fdgth.2024.1486662 (PMC11599239; doi:10.3389/fdgth.2024.1486662)
Supplement: Supplementary file 6 [file Table6.pdf]

Table S6. Result of the timed up and go test (12 months).

Variable	T0 (Mean ± SE)	T1 (Mean ± SE)	T2 (Mean ± SE)	*p-value*
**1. Spatio-temporal parameters**				
(a) Parameters for "sit to stand" and "stand to sit"				
Sit to stand				
Anterio-posterior acceleration, m/s2	3.81 ± 0.63	3.63 ± 0.37	3.32 ± 0.30	0.477 [b]
Lateral acceleration, m/s2	2.41 ± 0.72	2.19 ± 0.25	2.13 ± 0.45	0.236 [a]
Vertical acceleration, m/s2	4.48 ± 0.83	5.41 ± 0.52	6.12 ± 0.64	0.212 [b]
Stand to sit				
Anterio-posterior acceleration, m/s2	4.66 ± 0.58	4.46 ± 0.73	6.38 ± 1.10	0.219 [b]
Lateral acceleration, m/s2	2.93 ± 0.54	3.27 ± 0.77	3.89 ± 0.52	0.342 [b]
Vertical acceleration, m/s2	7.30 ± 0.85	6.72 ± 1.18	8.20 ± 1.49	0.440 [b]
(b) Parameters for "mid turning" and "end turning"				
Mid turning				
Maximum rotation speed, °/s	161.09 ± 22.19	147.76 ± 15.24	143.98 ± 17.09	0.394 [b]
Average rotation speed, °/s	81.62 ± 8.62	77.91 ± 6.07	77.53 ± 8.49	0.622 [b]
End turning				
Maximum rotation speed, °/s	170.53 ± 18.74	179.59 ± 18.67	186.93 ± 27.10	0.378 [b]
Average rotation speed, °/s	81.36 ± 13.68	97.32 ± 11.94	96.69 ± 14.60	0.196 [b]
**2. Phases durations**				
Sit to stand, s	1.39 ± 0.17	1.43 ± 0.13	1.58 ± 0.35	0.439 [a]
Forward gait, s	2.99 ± 0.55	2.34 ± 0.52	2.48 ± 0.58	0.641 [a]
Mid turning, s	2.62 ± 0.54	2.44 ± 0.20	2.77 ± 0.68	0.549 [a]
Return gait, s	2.75 ± 0.70	1.93 ± 0.22	1.53 ± 0.15	0.247 [a]
End turning - stand to sit, s	3.36 ± 0.75	2.46 ± 0.32	4.69 ± 2.59	0.293 [a]
Exam duration, s	14.03 ± 2.21	10.76 ± 1.14	13.33 ± 4.05	0.236 [a]

The values of T0, T1 and T2 are compared. *: $p < 0.05$ is considered as significant. a: Friedman's test. b: rANOVA. T0: at baseline, T1: after 6 months of intervention, T2: after 12 months of intervention, SE: standard error.
